# Aliskiren effect on non-alcoholic steatohepatitis in metabolic syndrome

**DOI:** 10.1186/s13098-017-0282-5

**Published:** 2017-10-13

**Authors:** F. N. Ramalho, S. C. Sanches, M. C. Foss, M. J. Augusto, D. M. Silva, A. M. Oliveira, L. N. Ramalho

**Affiliations:** 1 Department of Pathology and Legal Medicine, Faculty of Medicine of Ribeirão Preto, University of São Paulo, 14049-900 Ribeirão Preto, SP Brazil; 2 Department of Medicine, Faculty of Medicine of Ribeirão Preto, University of São Paulo, Ribeirão Preto, SP Brazil; 3 Department of Physics and Chemistry, Faculty of Pharmaceutical Sciences of Ribeirão Preto, University of São Paulo, Ribeirão Preto, SP Brazil

**Keywords:** Diabetes mellitus, Nonalcoholic steatohepatitis, Metabolic syndrome, Aliskiren

## Abstract

**Background:**

Non-alcoholic steatohepatitis (NASH) is highly associated with metabolic syndrome, a major cause of morbidity in the globalized society. The renin–angiotensin system (RAS) influences hepatic fatty acid metabolism, inflammation and fibrosis. Thus, in the present study, we aimed to evaluate the effect of aliskiren, a direct renin inhibitor, on metabolic syndrome-related NASH.

**Methods:**

C57BL/6 male mice (n = 45) were divided into three groups: controls; animals inoculated with streptozotocin (STZ) (40 mg/kg/day) for 5 days and fed with high fat diet (HFD) for 8 weeks; and animals inoculated with STZ for 5 days, fed with HFD for 8 weeks and treated with aliskiren (100 mg/kg/day) for the final 2 weeks. Glycemic and insulin levels, hepatic lipid profile, histological parameters and inflammatory protein expression were analyzed.

**Results:**

Aliskiren normalized plasma glucose and insulin levels, reduced cholesterol, triglycerides and total fat accumulation in liver and diminished hepatic injury, steatosis and fibrosis. These results could be explained by the ability of aliskiren to block angiotensin-II, lowering oxidative stress and inflammation in liver. Also, it exhibited a beneficial effect in increasing insulin sensitivity.

**Conclusion:**

These findings support the use of aliskiren in the treatment of metabolic syndrome underlying conditions. However, clinical studies are indispensable to test its effectiveness in the treatment of patients with metabolic syndrome.

## Background

Non-alcoholic fat liver disease (NAFLD) consists of a disease characterized by triglyceride accumulation in hepatocytes, due to causes not related with alcohol intake, resulting in progressive liver injury and fibrosis in the form of nonalcoholic steatohepatitis (NASH). NAFLD is highly associated with metabolic syndrome, a major cause of morbidity in the globalized society [1]. Metabolic syndrome can be defined as a compilation of medical disorders, for instance, obesity, impaired glucose tolerance, dyslipidemia and hypertension [2]. The challenge is to develop drugs that are able to act in a beneficial way in the treatment of these coexistent conditions.

Insulin resistance occurs in a high percentage of cases of NAFLD (66–83%) [3] and NAFLD is highly common (up to 70%) in type 2 diabetes [4], in which is observed increased hepatic triglyceride storage independently of obesity [5]. Insulin is known to increase sterol regulatory element binding proteins (SREBPs) in hepatocytes. SREBPs are transcription factors that modulate synthesis and uptake of cholesterol, fatty acids and triglycerides [6]. Moreover, insulin also regulates free fatty acid flux via fatty acid transport proteins (FATPs). Thus, in the absence of insulin, triglyceride is stored in the liver leading to steatosis [7]. Finally, insulin increases intrahepatic levels of the nuclear factor kappa-B (NF-kB), a transcriptional factor involved on pro-inflammatory response associated to NASH [8].

Renin, one of the pivotal components of renin–angiotensin system (RAS), is responsible for the conversion of the angiotensinogen to angiotensin-I (Ang-I). Angiotensin converting enzyme (ACE) hydrolyzes Ang-I to angiotensin-II (Ang-II). Most of Ang-II roles are mediated by Ang-II type 1 receptor (AT1R), such as vasoconstrictor, pro-inflammatory, pro-oxidative, proliferative and hypertrophic effects [9]. Angiotensin (1–7) (Ang 1–7) may be formed by ACE2, a homolog of ACE. Through its G-protein-coupled receptor Mas, Ang (1–7) induces vasodilation, anti-hypertrophic and anti-proliferative effects [10]. Activation of the ACE2/Ang (1–7)/Mas receptor axis could be associated with diminished insulin resistance by inducing the activation of insulin signaling pathways and counteracting the inhibitory effects of ACE/Ang-II/AT1R [11].

RAS also influences hepatic fatty acid metabolism via the activation of AT1R [12]. Several types of damage are reported to upregulate local RAS in the liver [13, 14]. RAS blockade attenuates hepatic inflammation and fibrosis by suppressing the activation of hepatic stellate cells (HSC) and oxidative stress [15, 16]. In addition, Ang-II receptor blockers (ARB) improve hepatic steatosis, fibrosis and inflammation in NASH models [17, 18]. In the liver, ARB also reduces oxidative stress products such as 4-hydroxynonenal (4-HNE), which is increased by insulin resistance and is associated to NASH progression [19].

Direct renin inhibitors (DRI) are drugs that reduce plasma renin activity (PRA), as well as local and circulating renin through interaction with the active site of the enzyme and hence reduce the formation of Ang-II [20]. Aliskiren was the first oral DRI utilized for treating hypertension. It binds to the active site of renin, reducing the ability of renin to convert angiotensinogen to Ang-I. This block, in the final routes, will restrict the synthesis of Ang-II. Thus, during the use of aliskiren, lower plasma renin activity is observed. This phenomenon occurs differently from ACE inhibitors and ARB, which increase plasma renin activity. In this case, there is probably a perception by the body of a decrease in the production or activity of Ang-II, generating a compensatory increase in the release of renin by the kidney, in an attempt to reestablish circulating levels of Ang-II. Using aliskiren, even if this compensatory mechanism is requested, there is a blockade already at the beginning of the RAS, preventing the production and activity of renin itself. In addition, since aliskiren acts by inhibiting renin, it is able to block RAS, without causing “leakage” to the production of Ang-II, which ACE inhibitors, beta-adrenergic receptor blockers and ARB may present. These “leaks” can occur for two main reasons: conversion of Ang-II from Ang-I by enzymes other than ACE, such as serine-like proteases, cathepsin G and cardiac chymase in the case of ACE inhibitors; and interaction of the AT1 and AT2 receptors with aldosterone, providing a stimulus on renin production in the case of ARB and ACE inhibitors, respectively [21, 22].

Aliskiren was also described as an effectiveness agent to restrain the Ang II deleterious properties in the liver. In a recent study, aliskiren reduced insulin resistance and steatosis in high fat diet-fed mice through stimulated fatty acid oxidation-related genes [23]. Previous studies have demonstrated the effect of aliskiren in a model of metabolic syndrome in fructose-fed rats [24].

However, the usefulness of aliskiren on NASH associated to metabolic syndrome was still unclear. Thus, this study aimed to evaluate the effect of aliskiren on NASH related to a model of diabetes/high fat diet-induced metabolic syndrome.

## Methods

### Animals

C57BL/6 mice (n = 45), at 8 weeks of age, weighing 20–25 g were housed under standard conditions, including temperature and a 12:12-h light/dark cycle, with food and water provided ad libitum. Animal management procedures conformed to the International Guiding Principles for Biomedical Research Involving Animals. All animal procedures were approved by the Ethics Committee for Animal Research (CETEA) of the School of Medicine of Ribeirão Preto, at University of São Paulo (nº0.15⁄ 2015). Efforts were made to minimize animal suffering and to reduce the number of animals used.

### Experimental design

Mice were randomly subdivided into the following three distinct groups: control group (C) (n = 15) was inoculated with intraperitoneal streptozotocin (STZ) vehicle and fed with standard diet; diabetes and high fat diet group (HDC) (n = 15) was inoculated with intraperitoneal STZ (Sigma-Aldrich Co., St. Louis, MO, USA) (40 mg/kg/day) for 5 consecutive days [25] and fed with a high fat diet rich in medium-chain fatty acid, composed of 36.8% fat, 1.25% cholesterol, 39.75% carbohydrates, 14.04% casein, 5.0% fiber, 1.0% vitamins, 1.6% minerals, 0.25% choline and 0.3% l-cysteine (5,5 kcal/g—Rhoster, Ind & Co, São Paulo, Brazil) ad libitum for 8 weeks [26]. For the control group, the standard diet is composed of 3.5 kcal/g—Rhoster, Ind & Co, São Paulo, Brazil). Aliskiren group (HDA) (n = 15) was inoculated with intraperitoneal STZ (Sigma-Aldrich Co.) (40 mg/kg/day) for 5 consecutive days and fed with a high fat diet rich in medium-chain fatty acid for 8 weeks. From the 6th week, mice received orally aliskiren (Novartis Pharma, Stein, Switzerland) (100 mg/kg/day) on alternate days for a period of 8 weeks [27]. For validation of STZ efficacy, plasma glucose levels were measured spectrophotometrically in serum using a commercial kit according to the manufacturer’s instructions (Labtest Inc., Lagoa Santa, MG, Brazil) at an absorbance of 505 nm. Mice were considered diabetic when glycaemia was above 250 mg/dl after two consecutive determinations [28].

Subsequently, mice were sacrificed under anesthesia with intramuscular ketamine (80 mg/kg) and xylazine (10 mg/kg). Fragments of liver tissue were fixed in 10% buffered formalin for 48 h and embedded in paraffin. Additional liver samples were also frozen in liquid nitrogen and stored at − 80 °C.

### Aminotransferases

Plasma alanine aminotransferase (ALT) and aspartate aminotransferase (AST) levels were measured spectrophotometrically in serum using a commercial kit according to the manufacturer’s instructions (Labtest, Lagoa Santa, Brazil) at an absorbance of 340 nm.

### Analysis of lipid profile

Plasma triglycerides and total cholesterol were quantified spectrophotometrically in serum using a commercial kit according to the manufacturer’s instructions (Labtest, Lagoa Santa, Brazil) at an absorbance of 505 nm.

### Measurement of hepatic fat content

Part of the frozen liver samples (100 mg) were homogenized and hepatic lipid content of the samples was extracted with chloroform and methanol (2:1) [29].

### Histological analysis

Formalin-fixed and paraffin-embedded tissue sections were cut to a thickness of 5 μm and stained with hematoxylin and eosin for histological examination. A pathologist, who was blinded to the treatment, used light microscopy to assess the percentage of animals with liver steatosis. Analysis was performed on 30 randomly chosen high-power fields (HPFs; × 400 magnification) in each slide. Grading of the severity of hepatic steatosis was as follows: grade 0, minimal or no evidence of steatosis < 5%; grade 1, mild steatosis 6–33%; grade 2, moderate to severe steatosis 34–66%; and grade 3, severe steatosis > 66%. The portal and lobular inflammation was also scored as follows: grade 0, minimal or no evidence of inflammation; grade 1, mild inflammation; grade 2, moderate to severe inflammation; and grade 3, severe inflammation [30]. The percentage of liver steatosis was also assessed using Sudan III staining. The steatosis amount hepatic steatosis was measured as the percentage of Sudan III-positive staining (orange) hepatocytes in 30 random HPFs (× 400 magnification) using Image J software (Image J, 1.33u, NIH, USA). Infiltration of neutrophils into the liver was estimated using the naphthol AS-D chloroacetate esterase staining method, which identifies specific leukocyte esterases. Briefly, the 5 µm paraffin sections were deparaffinized with xylene, rehydrated through an alcohol series, and then immersed in distilled water before being incubated in a naphthol esterase solution at room temperature for 15 min. Naphthol esterase solution contains naphthol AS-D chloroacetate (Sigma Chemical Co., St. Louis, MO, U.S.A.) in N,N-dimethyl formamide (2 mg/mL), 4% sodium nitrite, and 4% new fuchsin in 2 N HCl combined in 0.1 M phosphate buffer (pH 7.6). Then, the sections were rinsed with tap water and counterstained with Gill’s hematoxylin for 15 s. Red color was deposited only in the neutrophils and mast cells. The identification of the stained neutrophils was made based on the nuclear morphology and appearance of small red granular deposits scattered within the cytoplasm. Only polymorphonuclear cells (PMN) located within sinusoids or extraverted into the surrounding parenchyma and characterized with a multi-lobed nucleus and red granular deposits within the cytoplasm were counted. The number of esterase-positive PMN was counted in 30 HPFs (× 400 magnification) in each sample, and the mean values were calculated. Additional slides were stained using Sirius Red. Collagen deposition was measured as the percentage of Sirius Red-positive staining (red) cells in 30 random HPFs (× 400 magnification) using Image J software (Image J, 1.33u, NIH, USA).

### Immunohistochemical detection of 4-hydroxinonenal adducts

Further liver preparations were also submitted for immunohistochemical analysis. Briefly, 4-μm-thick sections mounted on poly-l-lysine-coated slides were deparaffinized, rehydrated, immersed in 10 mmol/L citrate buffer (pH 6.0) and subjected to heat-induced epitope retrieval using a vapor lock for 45 min. Slides were rinsed with phosphate-buffered saline (PBS) and immersed in 3% hydrogen peroxide for 20 min to block endogenous peroxidases. Non-specific protein binding was blocked with normal serum (Vectastain Elite ABC Kit, Universal, Vector Laboratories Inc., Burlingame, CA, USA) for 30 min. Sections were then incubated with a monoclonal primary antibody specific for monoclonal anti-4-hydroxinonenal (4-HNE) adduct antibody (A.G. Scientific Inc., San Diego, CA, USA), diluted 1:100 for 2 h at room temperature (25 °C) in a humid chamber. Following washes in PBS, a biotinylated pan-specific universal secondary antibody (Vectastain Elite ABC Kit, Universal) was applied for 30 min. Next, slides were incubated with avidin–biotin–peroxidase complex (Vectastain Elite ABC Kit, Universal) for 30 min and developed using NovaRed kit (Vector Laboratories Inc.) for 5 min. Slides were counterstained with Harris’s hematoxylin, dehydrated and mounted with Permount (Biomeda Co., Foster City, CA, USA). For negative controls, all specimens were incubated with an isotope-matched control antibody under identical conditions. Analysis was performed on 30 randomly chosen HPFs (× 400 magnification). Immunoreactivity to 4-HNE was evaluated as the percentage of positive labeling/HPF using Image J software (Image J, 1.33u, NIH, USA).

### Southwestern histochemistry analysis

The non-radioactive in situ detection of NF-kB in paraffin-embedded liver tissue preparations was performed using the Southwestern Histochemistry method, with digoxigenin labeling and detection kits (Roche Applied Science, Indianapolis, USA). Briefly, synthetic sense DNAs (Imprint Genetics Corporation, Hialeah, USA), which contain sequences of NF-kB, were used as probes. After annealing with the complementary sequence, the DNA probe was labeled with digoxigenin. The sections were then incubated with the labeled probes for 12 h at 37 °C. The slides were then incubated with an anti-digoxigenin antibody conjugated with alkaline phosphatase and detected using a NBT/BCIP solution. The slides were then mounted with glycerol. The mutant form of the probe labeled with digoxigenin was used as a negative control. NF-kB labeling was considered to be positive when distinct purple nuclear staining was present homogenously. For negative controls, all specimens were incubated with an isotope-matched control probe under identical conditions. Analysis was performed on 30 randomly chosen HPFs (× 400 magnification). Immunoreactivity to NF-kB was evaluated as the percentage of positive labeling/HPF using Image J software (Image J, 1.33u, NIH, USA) [31].

### Detection of IR1, NF-kB, pAKT1/2/3 (Ser 473) and MasR by western blotting

Additional frozen liver Sects. (100 mg) were homogenized in 1 mL of lysis buffer [20 mM EDTA, 1% Triton, 0.1% SDS, 10 mM NaF, 1 mM Na_3_VO_4_, 10 mM glycerophosphate and protease inhibitors (one tablet per 10 mL buffer, Complete, Roche Applied Science, Mannheim, Germany)] for 30 s. Suspensions were incubated on ice for 15 min and centrifuged at 10,000 rpm at 4 °C for 20 min. Protein concentrations were measured using Bradford colorimetric method. Then, 50 μg protein were resolved by electrophoresis on 10% SDS-polyacrylamide gels, and blotted onto PVDF membranes (Amersham Life Science, Arlington Heights, IL, USA). After blocking with TBS solution (pH 7.4) containing 0.05% Tween 20 and 5% fat free milk for 1 h at room temperature, membranes were incubated “overnight” at 4 °C with primary monoclonal antibodies to insulin receptor type 1 (Santa Cruz Biotechnology Inc., Dallas, Texas, USA; 1:500 dilution), NF-kB-p65 (Santa Cruz Biotechnology Inc.; 1:500 dilution), pAKT1/2/3(Ser 473)(Santa Cruz Biotechnology Inc.; 1:200 dilution), Mas-Receptor (Alomone Laboratories, Jerusalem, Israel; 1:500 dilution) and GAPDH (Sigma-Aldrich Co.; 1:1000 dilution). Bands were detected using a chemiluminescence system with HRP-conjugated secondary antibodies and ECL-Plus reagents (G&E Healthcare Life Sciences, Little Chalfont, Buckinghamshire, UK). Molecular weight markers were used to determine protein size (Sigma-Aldrich Co.), and GAPDH was used as an internal control. Resulting blots were scanned with ImageQuant LAS 4000 (G&E Healthcare Life Sciences). Relative densities of the bands were analyzed and quantified with ImageQuant TL software (G&E Healthcare Life Sciences).

### Gene expression by real time PCR

The liver samples stored at − 80 °C were also used for the quantification of the gene expression of Interleukin 1-β and TNF-α. The tissue samples, homogenized with the aid of a Polytron homogenizer (PT 2100, Kinematica Polytron, Newark, NJ, USA) were subjected to extraction of total RNA using refrigerated centrifuge (Mikro 220R Hettich Zentrifugen, Germany) and specific kit (RNAqueous^®^-4PCR DNA-freeTM RNA Isolation for RT-PCR). The complementary DNA was obtained by reverse transcription from using retrotranscription kit (Ominiscript^®^ RT Kit, Qiagen). The simultaneous quantification in gene amplification was performed by employing StepOnePlus™ (Applied Biosystems, CA) using primers specific for the genes of Interleukin 1-β and TNF-α and 18S (Assayson-Demand Gene Expression Products, Applied Biosystems, Foster City, CA) and Taq Polymerase (TaqMan Universal PCR Master Mix, No AmpErase UNG-2X Applied Biosystems). There was employed for the analysis of ΔΔCT method from the difference between samples using the gene 18S reference.

### Statistical analysis

Data were analyzed using GraphPad Prism software 4.0 (GraphPad Software, San Diego, CA, U.S.A.). All data are reported as mean ± S.E.M. Statistical comparisons of the groups were performed by nonparametric Kruskal–Wallis one-way analysis of variance followed by Dunn’s posttest or Mann–Whitney test. Probability values less than 0.05 were considered to be statistically significant.

## Results

**Fig. 1:**
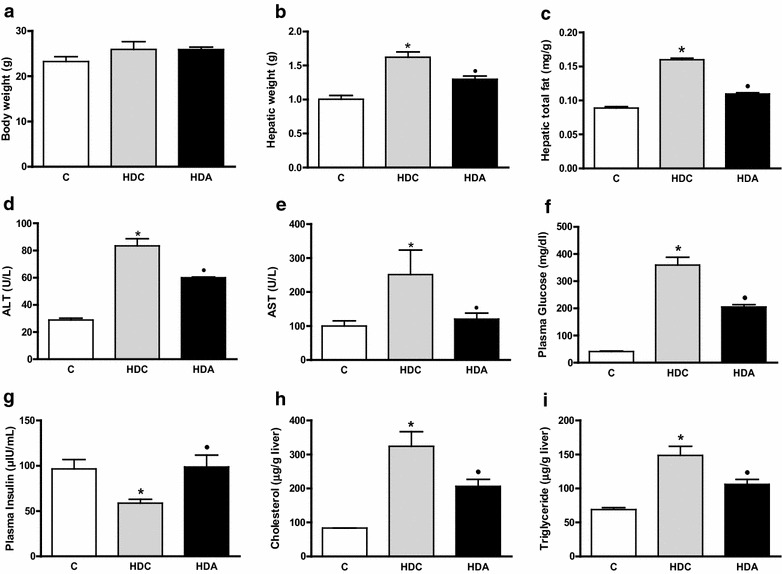
Body weight (**a**); hepatic weight (**b**); hepatic total fat (**c**); ALT dosage (**d**); AST dosage (**e**); plasma glucose (**f**); plasma insulin (**g**); total cholesterol (**h**) and triglycerides (**i**) deposition in the liver in the control group (C); diabetic and high fat diet mice (HDC); diabetic and high fat diet mice treated with aliskiren (HDA). *P < 0.05, C × HDC; ^●^P < 0.05, HDC × HDA. (*n* = 4–6 mice per group)

Mice from control group (C) (23.2 ± 1.12), diabetes and high fat diet group (HDC) (25.9 ± 1.73) and aliskiren treatment (HDA) (25.9 ± 0.53) were similar in regard to body weight (Fig. 1a). HDC showed augmented hepatic weight (1.6 ± 0.07; P < 0.01) (Fig. 1b), hepatic total fat (0.16 ± 0.002; P < 0.05) (Fig. 1c), ALT dosage (83.3 ± 5.31; P < 0.01) (Fig. 1d) and AST dosage (251.5 ± 71.97; P < 0.05) (Fig. 1e) when compared to C (1.0 ± 0.05; 0.089 ± 0.001; 28.75 ± 1.49; 100.0 ± 15.42; respectively). Aliskiren treatment diminished hepatic weight (1.3 ± 0.04; P < 0.01) (Fig. 1b), hepatic total fat (0.10 ± 0.001; P < 0.05) (Fig. 1c), ALT dosage (60.0 ± 0.58; P < 0.01) (Fig. 1d) and AST dosage (120 ± 17.55; P < 0.05) (Fig. 1e). HDC presented increased plasma glucose (359.6 ± 28.5) when compared with C (41.4 ± 1.9; P < 0.01). Aliskiren treatment decreased plasma glucose (205.0 ± 9.1; P < 0.01) (Fig. 1f). Additionally, aliskiren (98.7 ± 13.1) reversed HDC plasma insulin levels (58.9 ± 4.1) to values similar to control (96.5 ± 10.5; P < 0.05) (Fig. 1g). Cholesterol deposition in liver was lowered in HDA group (206.2 ± 21.1) when compared with HDC group (324.6 ± 42.6; P < 0.05) (Fig. 1h), reaching levels similar to the control group (225.9 ± 7.4). Similarly, triglycerides liver storage was reduced in HDA group (106.1 ± 7.2) when compared with HDC group (148.8 ± 13.3) which was augmented in comparison to the control group (109.0 ± 17.3; P < 0.05) (Fig. 1i).

**Fig. 2:**
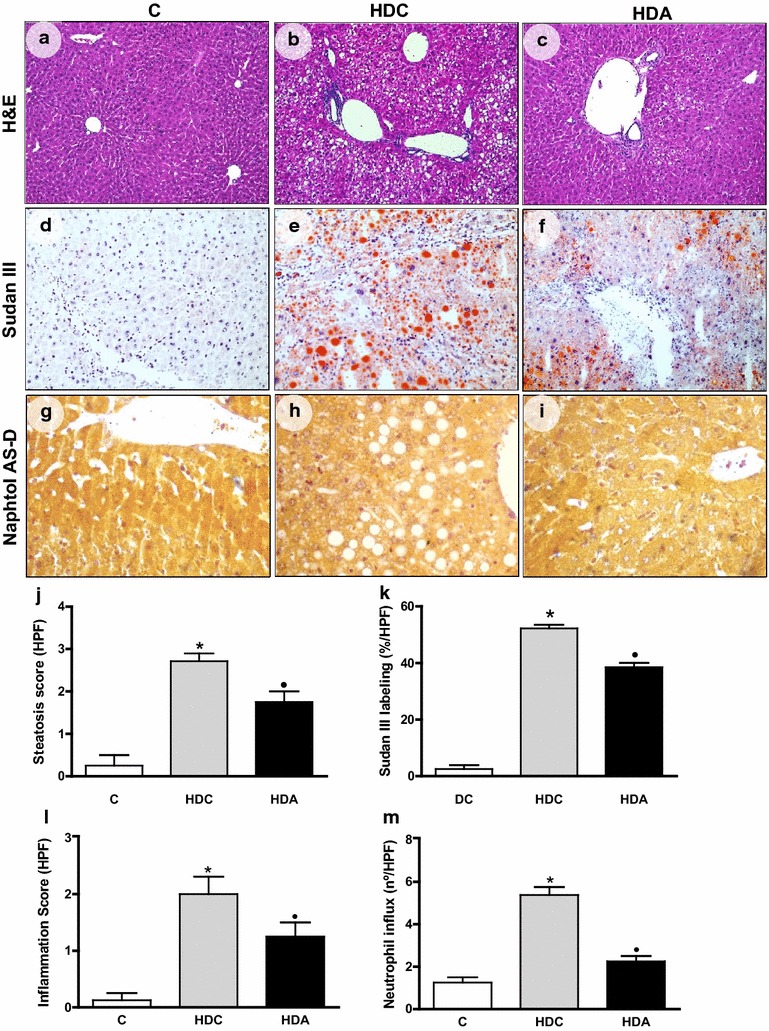
Representative photomicrographs of liver sections stained with Hematoxylin & Eosin (**a**–**c**), Sudan III (**d**–**f**) and Naphtol AS-D (**g**–**i**) in the liver in the control group (C); diabetic and high fat diet mice (HDC); diabetic and high fat diet mice treated with aliskiren (HDA) (× 400 magnification). Graphic representation of steatosis score (**j**), Sudan III labeling (**k**), inflammation score (**l**) and neutrophil influx (**m**) in C, HDC and HDA groups. *P < 0.05, C × HDC; ^●^P < 0.05, HDC × HDA. (*n* = 4–6 mice per group)

**Fig. 3:**
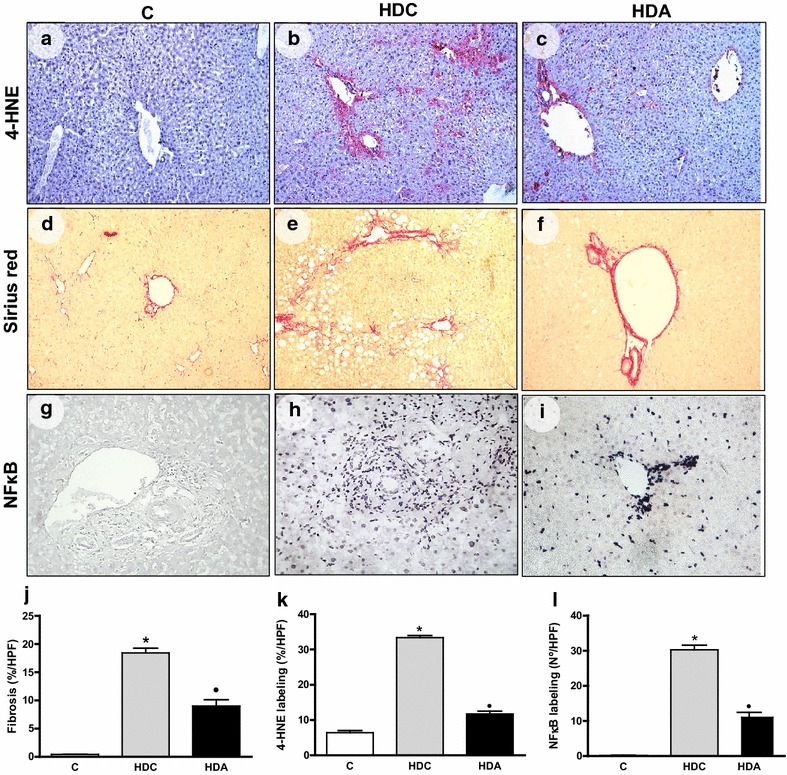
Representative photomicrographs of liver sections stained with 4-HNE (**a**–**c**), Sirius red (**d**–**f**) and NF-kB (**g**–**i**) staining in the liver in the control group (C); diabetic and high fat diet mice (HDC); diabetic and high fat diet mice treated with aliskiren (HDA) (× 400 magnification). Graphic representation of fibrosis (**j**), 4-HNE labeling (**k**) and NF-kB labeling (**l**) in C, HDC and HDA groups. *P < 0.05, C × HDC; ^●^P < 0.05, HDC × HDA. (*n* = 4–6 mice per group)

The photomicrographs show steatosis distribution (H&E) (Fig. 2a–c), lipid disposal (Sudan III) (Fig. 2d–f), neutrophil influx (Naphtol AS-D) (Fig. 2g–i), 4-HNE labeling (Fig. 3a–c), collagen deposition (Sirius red) (Fig. 3d–f) and NF-kB staining (Fig. 3g–i) in liver samples from the control, HDC and HDA groups. In H&E and Sudam III stain, enhanced steatosis and lipid deposition, respectively, were showed in HDC group (2.71 ± 0.18, P < 0.05; 52.29 ± 1.22, P < 0.05, respectively) when compared with C group (0.25 ± 0.25; 2.5 ± 1.44, respectively). Reduction in steatosis levels and lipid deposition were observed after aliskiren treatment (1.75 ± 0.25, P < 0.05; 38.50 ± 1.55, P < 0.05) (Fig. 2j, k). In Naphtol AS-D stain, increased inflammation and neutrophil influx were observed in HDC group (2.00 ± 0.30, P < 0.05; 5.37 ± 0.37, P < 0.01, respectively) when compared with C group (0.12 ± 0.12; 1.25 ± 0.25, respectively). Aliskiren treatment reversed these results (1.25 ± 0.25, P < 0.05; 2.25 ± 0.25, P < 0.01) (Fig. 2l, m).

The 4-HNE labeling percentage was higher in the HDC group (33.4 ± 0.6) than the C group (6.4 ± 0.5) (P < 0.05), but HDA group (11.7 ± 0.7) presented with a diminished 4-HNE labeling relative to the HDC group (P < 0.05) (Fig. 3 a–c, k). The collagen deposits were augmented in HDC group (18.4 ± 0.8) relative to the control group (0.4 ± 0.1) (P < 0.05). Aliskiren (9.0 ± 1.1) reduced collagen levels when compared with HDC group (P < 0.01) (Fig. 3d–f, j). The NF-kB labeling percentage was superior in the HDC group (30.29 ± 1.26) in relation to the control group (0.17 ± 0.06) (P < 0.05). While in HDA group, it was observed decreased values (11.00 ± 1.41) when compared to HDC group (P < 0.01)(Fig. 3g–i, l).

**Fig. 4:**
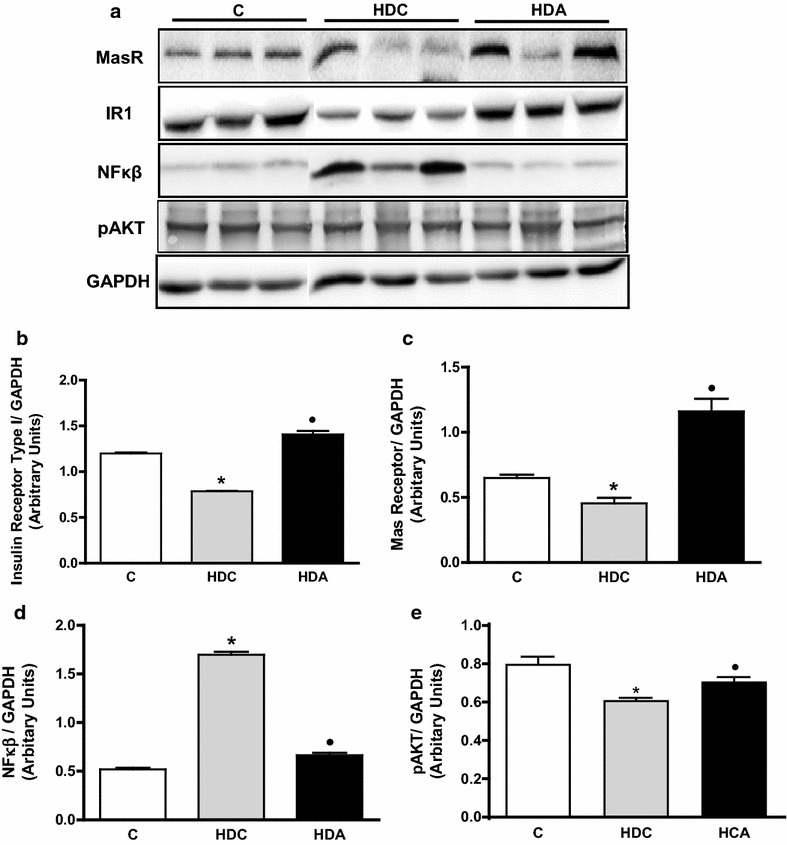
Images of insulin receptor type I (IR1), Mas receptor (MasR), nuclear factor kappa B (NF-kB) and pAKT protein quantification by Western blotting in the liver in the control group (C); diabetic and high fat diet mice (HDC); diabetic and high fat diet mice treated with aliskiren (HDA) (**a**). Graphic representation of IR1 (**b**), MasR (**c**), NF-kB (**d**) and pAKT (**e**) protein quantification in C, HDC and HDA groups. *P < 0.05, C × HDC; ^●^P < 0.05, HDC × HDA. (*n* = 4–6 mice per group)

Aliskiren treatment enhanced insulin receptor type I (IR1) and pAKT protein amount (1.4 ± 0.1; 0.7 ± 0.02, respectively) in relation to HDC group (0.8 ± 0.1, P < 0.01; 0.6 ± 0.01 P < 0.05, respectively), at levels similar to the controls (1.2 ± 0.1; 0.8 ± 0.03, respectively) (Fig. 4a, b, e). HDC group presented reduced Mas receptor protein amount (0.5 ± 0.1) in relation to C (0.6 ± 0.1) (P < 0.01). Aliskiren treatment augmented Mas receptor protein amount (1.2 ± 0.1) (P < 0.01) (Fig. 4a, c). Aliskiren treatment reduced NF-kB protein amount (0.7 ± 0.1) in relation to HDC group (1.7 ± 0.0) (P < 0.01) reaching the control values (0.5 ± 0.1) (Fig. 4a, d).

**Fig. 5:**
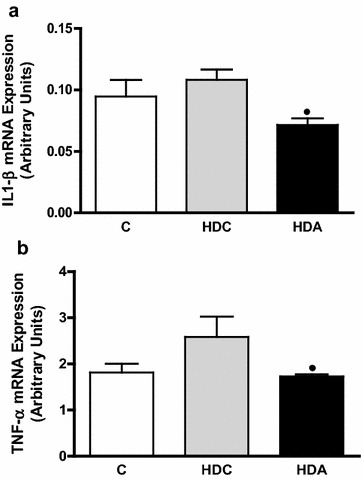
Graphic representation of IL-1β (**a**) and TNF-α (**b**) mRNA expression in the liver in the control group (C); diabetic and high fat diet mice (HDC); diabetic and high fat diet mice treated with aliskiren (HDA). ^●^P < 0.05, HDC × HDA. (*n* = 4–6 mice per group)

Finally, aliskiren treatment also prevented increased IL-1β and TNF-α gene expression observed in HDC group (0.07 ± 0.005 vs. 0.1 ± 0.008, P < 0.05; 1.73 ± 0.04 vs. 2.58 ± 0.44, P < 0.05, respectively) (Fig. 5a, b).

## Discussion

Aliskiren is a direct rennin inhibitor, which main aim is to prevent the formation of angiotensin I by blocking renin from converting to angiotensin I [20]. In this present study, aliskiren, besides its antihypertensive activity, exhibited effects on NASH associated to metabolic syndrome. Aliskiren normalized plasma glucose and insulin levels, reduced hepatic total fat, cholesterol and triglycerides accumulation, thus diminishing steatosis, oxidative stress and fibrosis in liver.

ARB were described as important options to reduce hepatic steatosis, fibrosis and inflammation in NASH models [17, 18], and to diminish oxidative stress associated to insulin resistance during NASH progression [19]. However ARB may act only partially in the reversion of NASH in obese and insulin resistant mice [32]. Perhaps, the effects of these drugs may be mitigated by the negative feedback loop and increased renin. On the other hand, aliskiren has been proved as an effective way to block renin activity and restrain liver steatosis and fibrosis in different models of murine NASH [33–35].

In the present model, we have demonstrated that diabetes associated with a high fat diet elicited the elevation of glucose, ALT and AST dosage in plasma. Moreover, hepatic total fat, triglycerides and total cholesterol deposition were enhanced, explaining augmented hepatic weight in HDC group. These findings were consistent with previous reports of metabolic syndrome proposal [36]. In addition, we found classical histological NASH pattern in the liver [30].

Our findings demonstrated that aliskiren treatment blocked the storage of cholesterol, triglycerides and total fat in liver, therefore diminishing the organ weight. H&E and Sudam III staining reinforced these results, as expected. Similarly, aliskiren diminished ALT and AST dosage in plasma, which was accompanied by a reduction in the influx of neutrophils as was observed by Naphtol AS-D labeling.

Furthermore, aliskiren interrupted the increase of glucose and stimulated the rise of plasma insulin levels when compared to the diabetes/high fat diet group. This result is supported by studies demonstrating that aliskiren triggers insulin secretion from beta cells [37]. The decreased plasma glucose after aliskiren treatment can be explained by its ability to increase insulin secretion or enhance insulin sensitivity, proved by the rise in the expression of insulin receptor type I and pAKT—a key downstream insulin effector—in the group treated with aliskiren. Gandhi et al. also explained aliskiren effects in insulin sensitivity by an upregulation in liver and muscle glucotransporter expression levels [38]. Moreover, Kang et al. found an improvement in insulin resistance, lipid abnormality and fibrosis in target organs in db/db mice after aliskiren treatment. They explained this effect by the significant increase in insulin sensitivity [39].

Previous studies have shown that aliskiren reduces NASH in mice fed with a high fat diet [23]. However our study was the first to demonstrate that aliskiren is able to attenuate NASH and ameliorate lipid and glycemic profile in a model of diabetes/high fat diet-induced metabolic syndrome. We attribute these findings to the changes in lipid metabolism, oxidative stress and cytokine expression elicited by aliskiren treatment. Lee et al. demonstrated an increased hepatic turnover of triglycerides with an upregulation in fatty acid transport and breakdown after aliskiren treatment [33].

Our findings demonstrated a reduction in 4-HNE. Bataller et al. elucidated the role of Ang-II in p47 phox phosphorylation and rise of reactive oxygen species in the liver [40].Wei et al. showed that in transgenic Ren2 rat model, Ang-II contributed to the progression of NAFLD by increasing hepatic reactive oxygen species [41]. Oxidative stress induces the progression of NASH through stimulation of inflammatory response [42]. The production of proinflammatory cytokines, such as TNF-α, IL-1β and NF-kB and infiltration of inflammatory cells may be diminished subsequently to the inhibition of Ang-II and reactive oxygen species in the liver [43, 44]. Thus, aliskiren have caused the decrease of Ang-II and consequently diminished oxidative stress and inflammatory activity in the liver.

We also observed a decrease in liver fibrosis in the mice receiving aliskiren. Hepatic fibrosis is mainly characterized by enhanced deposition of collagen in liver. Ang-II stimulates reactive oxygen species and consequently promotes liver inflammation and progression to fibrosis, through the activation of HSC. Furthermore, oxidative stress triggers hepatocyte apoptosis, increasing liver injury, which facilitates the fibrogenic process [40, 45]. Aliskiren may have caused a decrease in Ang-II and therefore, in liver fibrosis.

Finally, Ang-II is converted to Ang (1–7) by ACE2. Ang (1–7) plays a role in antagonizing ng-II effects through Mas receptor. Ang (1–7) levels and its therapeutic benefits are neutralized after aliskiren treatment [46]. In our experiments, after aliskiren treatment, we observed an increase in Mas receptor indirectly reflecting the decrease in Ang (1–7), since once it is decreased, there is a signal for a greater expression of its receptor. This finding reinforces the effect of aliskiren on the inhibition of renin. The lower production of Ang (1–7) observed was not sufficient to counteract the important effects of Ang-II inhibition on hepatic fibrosis and inflammation in the present steatohepatitis model.

In conclusion, by blocking Ang-II and thus oxidative stress and inflammation, aliskiren attenuates liver inflammation, steatosis and fibrosis. Additionally, it increases plasma insulin levels and insulin sensitivity. Thus, aliskiren is a promising drug in the treatment of metabolic syndrome underlying conditions. However, clinical studies are indispensable to test its effectiveness in the treatment of patients with metabolic syndrome.

## Abbreviations

NAFLD: non-alcoholic fat liver disease
NASH: nonalcoholic steatohepatitis
SREBPs: sterol regulatory element binding proteins
FATPs: fatty acid transport proteins
NF-κB: nuclear factor kappa-B
RAS: renin–angiotensin system
Ang-I: angiotensin-I
ACE: angiotensin converting enzyme
Ang-II: angiotensin-II
AT1R: ang-II type 1 receptor
Ang 1-7: angiotensin (1-7)
HSC: hepatic stellate cells
ARB: ang-II receptor blockers
4-HNE: 4-hydroxynonenal
DRI: direct renin inhibitors
STZ: streptozotocin
ALT: alanine aminotransferase
AST: aspartate aminotransferase
